# Bilateral Osteonecrosis of the Femoral Head During Pregnancy Following Two Corticosteroid Injections: A Case Report and Review of the Literature

**DOI:** 10.7759/cureus.556

**Published:** 2016-04-03

**Authors:** Thomas J Wood, Daniel j Hoppe, Mitchell Winemaker, Anthony Adili

**Affiliations:** 1 Division of Orthopaedic Surgery, McMaster University; 2 Department of Orthopaedic Surgery, Stanford University; 3 Division of Orthopaedic Surgery, Juravinski Hospital, McMaster University; 4 Division of Orthopaedic Surgery, St. Joseph's Hospital, McMaster University

**Keywords:** osteonecrosis, avascular necrosis, pregnancy, steroids, femoral head

## Abstract

Osteonecrosis of the femoral head during pregnancy, or shortly thereafter, is a rare clinical problem. Little is known about pregnancy as an etiological factor for femoral head osteonecrosis with only 40 reported cases in the literature. Furthermore, single or dual dose steroid-induced multifocal osteonecrosis is a controversial topic with only a handful of published cases. We present a case of a 34-year-old female with bilateral femoral head osteonecrosis that developed during the peripartum period. She received two large intramuscular injections of steroids for fetal lung maturity because early delivery was required as a result of eclampsia. She underwent total arthroplasty of the left hip due to unremitting pain and functional disability, which achieved good clinical results--relieving her pain and improving her range of motion. Literature is scarce with regard to single or dual dose steroid-induced osteonecrosis of the hip as well as pregnancy as a general etiologic factor. This case highlights the need for high clinical suspicion of osteonecrosis as a cause of postpartum hip pain.

## Introduction

Osteonecrosis (ON) is believed to occur due to a loss of blood supply to the affected subchondral bone [1]. The femoral head is particularly prone to ON due to its limited collateral circulation, accounting for 20,000 cases per year in the United States [2-3]. The average age at presentation ranges from 30 to 50 years, with known associations with hyperlipidemia, alcoholism, Cushing’s syndrome, hyperuricemia, sickle cell anemia, lupus, and rheumatoid arthritis [2-4]. A particularly devastating cause is steroid use, which may result in fat and/or thromboembolism and compromise the blood supply to the femoral head [1]. However, little is understood about the duration and quantity of steroids required to cause osteonecrosis [5-7].

Furthermore, osteonecrosis of the femoral head during or just after pregnancy is a rare clinical problem, reported for the first time by Pfeifer in 1957 [8]. Little is known about pregnancy as an etiological factor for femoral head osteonecrosis with approximately 40 reported cases in the literature [9-11]. The purpose of this case is to highlight the importance of osteonecrosis as a cause of postpartum hip pain and to review the literature with regard to pregnancy and single or dual dose steroids as etiological factors. 

## Case presentation

A 34-year-old female was seen in our outpatient clinic in November 2008 for assessment of bilateral hip pain. Her pain began in July 2008, during the end of her first pregnancy, which required an expedited delivery by caesarian section due to the development of eclampsia. She received two intramuscular injections of 12 mg of betamethasone (prednisone equivalence of 75 mg) over a 48 hour period for fetal lung maturity prior to delivery. Her pain developed immediately following the delivery, two days after receiving the first injection. She subsequently developed hip pain, worse on the left side, which was progressive in nature and radiated down her anterior thigh and knee. It was aggravated from weight bearing, with her functional status limited to walking the length of her driveway.

Her past medical history was significant for hypothyroidism and bilateral ACL reconstruction. She was taking Synthroid and reported no allergies.

On examination, she had a notable antalgic gait favouring the left side, with one centimeter of shortening. Range of motion of the left hip was limited from 0-80 degrees of flexion, 0 degrees of internal rotation, and external rotation and abduction to approximately 20 degrees. The left knee exam was normal. The right hip had mild discomfort with full range of motion, aside from a mild decrease in internal rotation.

**Figure 1:**
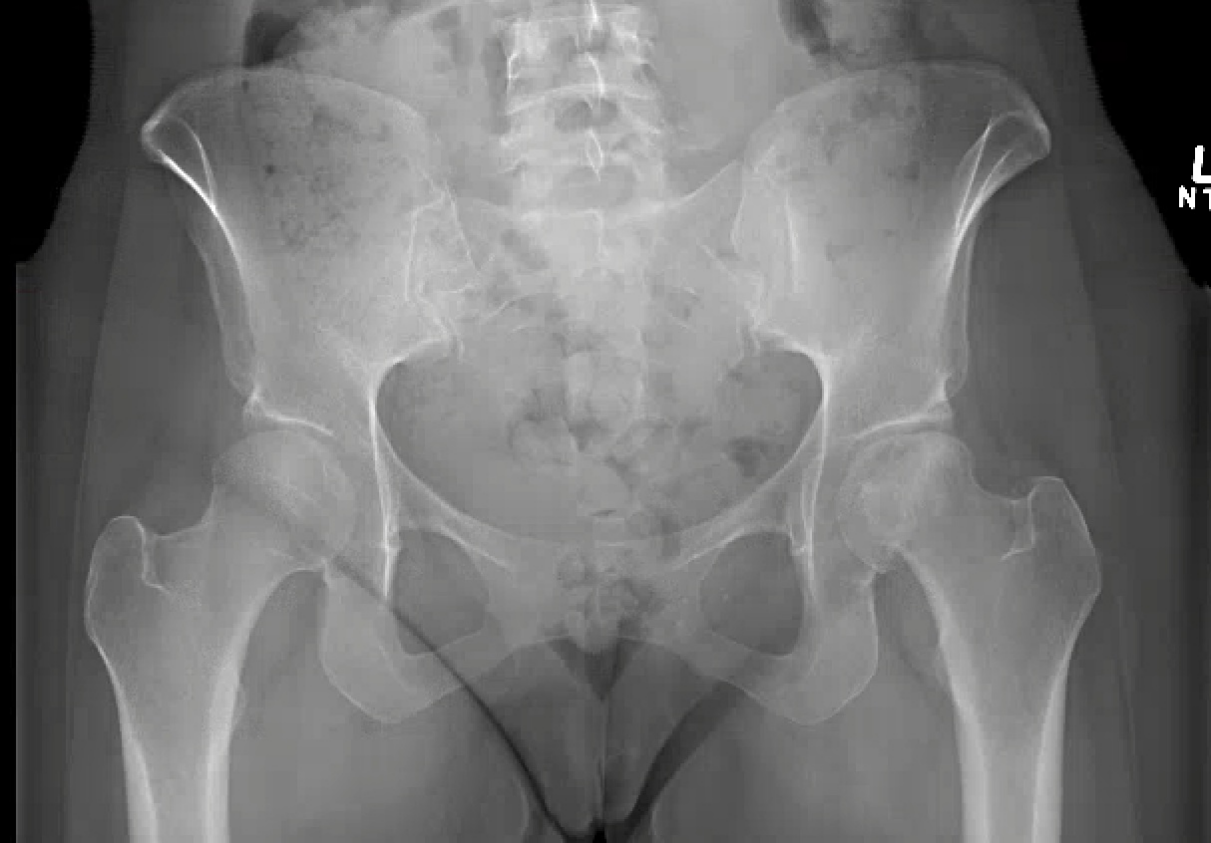
Initial anteroposterior radiograph of the pelvis showing left femoral head sclerosis and collapse, with minimal joint space narrowing.

**Figure 2:**
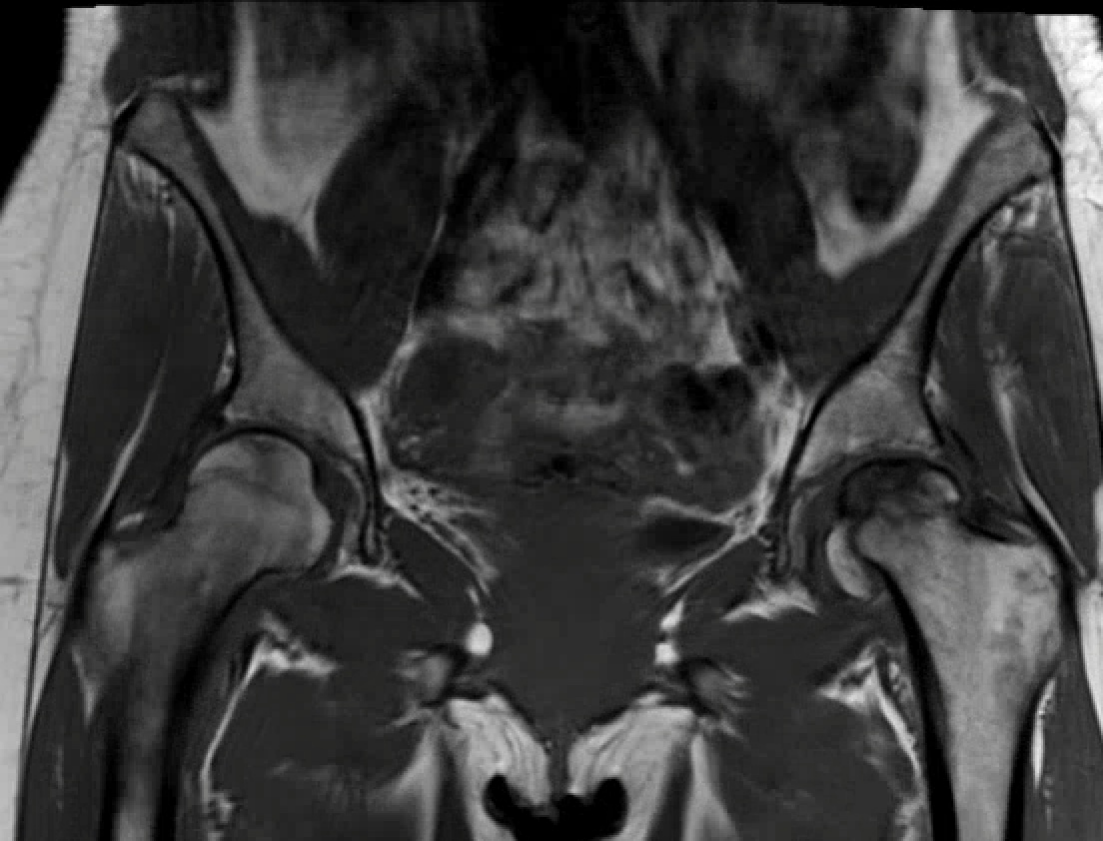
Coronal T2-weighted magnetic resonance imaging of both hips showing collapse of the left femoral head, and a focal area of osteonecrosis in the right femoral head.

Radiographs of the left hip showed collapse and sclerosis of the femoral head with preservation of the joint space, while x-rays of the right hip were unremarkable (Figure 1). An MRI of the left hip showed significant osteonecrosis with collapse of the femoral head and abnormal articulation (Figure 2). The MRI of the right hip revealed a small area of osteonecrosis in the superolateral head with no collapse. This imaging was in keeping with Ficat Grade 4 osteonecrosis on the left and Grade 2 on the right [12].

**Figure 3:**
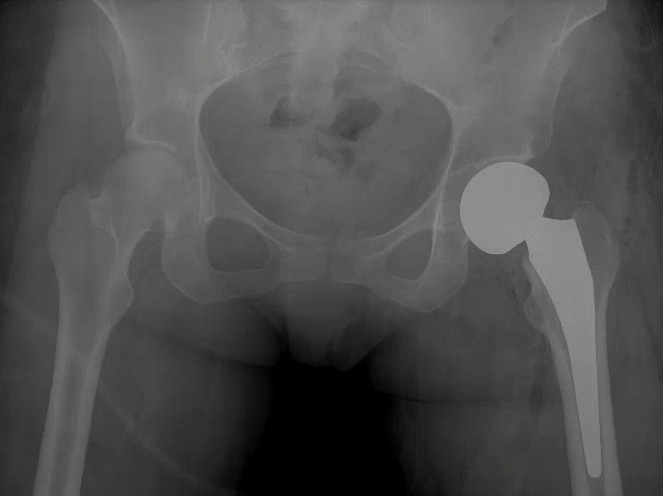
Immediate post-operative radiograph after the patient underwent a left hip hemiarthroplasty.

Given her symptoms and degree of immobility, it was recommended that a hemiarthroplasty be performed. This was performed in April 2009 using an Accolade 2.5-132 femoral press fit prosthesis, a minus 4V collar, and with a 49 mm unitrax unipolar ball (Stryker). In addition, debridement of the labrum was performed. She tolerated this procedure well, and there were no complications (Figure 3).

Unfortunately, she presented one year later with recurrent bilateral hip pain, again left worse than right, which was activity related and radiated to her knees. She was taking Meloxicam and regularly attending physiotherapy. On examination, she had symmetric range of motion of both her hips, with no limitations in internal or external rotation. However, she experienced deep anterior pain in her left hip with terminal flexion.

**Figure 4:**
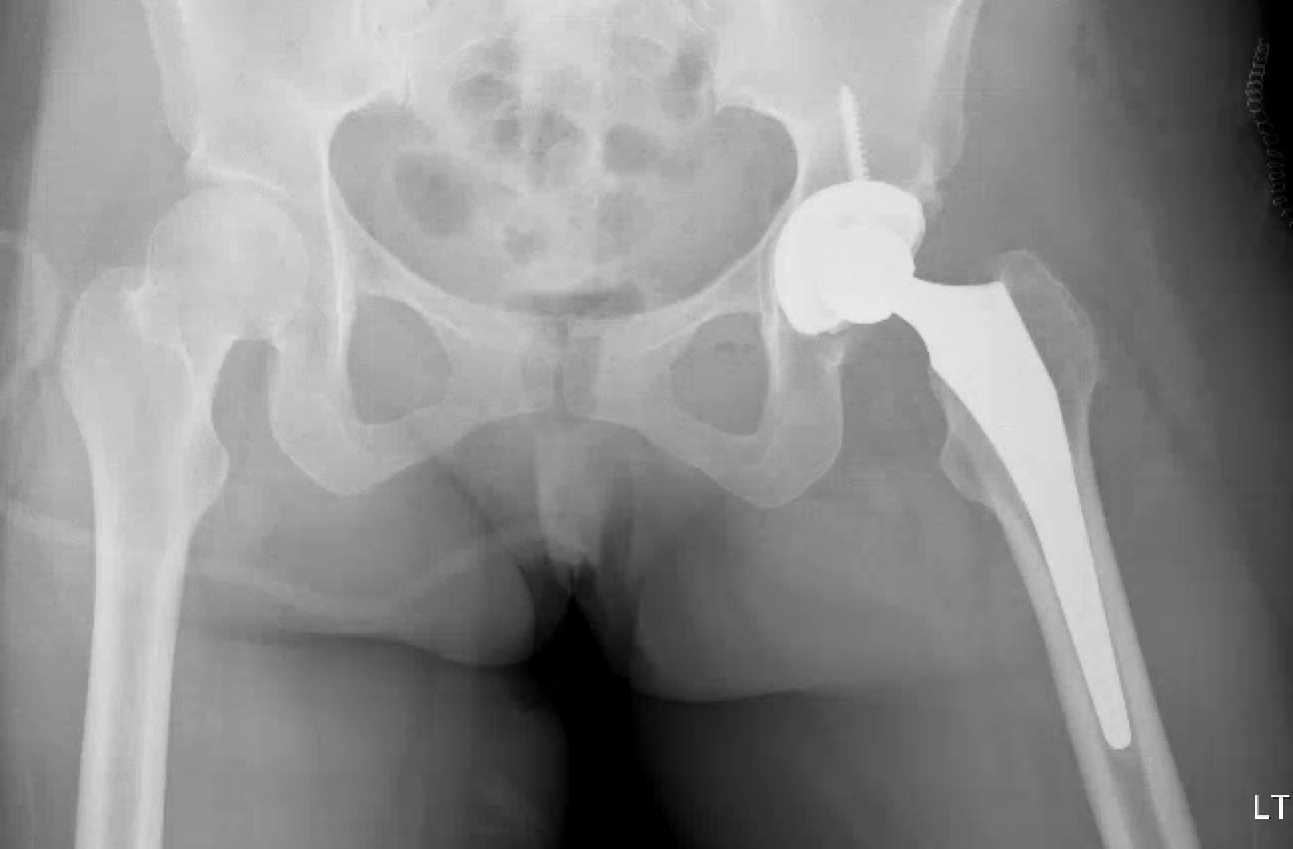
Post-operative radiograph after the patient underwent conversion to an uncemented left total hip arthroplasty.

Radiographs performed at this time demonstrated no interval changes on either side. The stem on the left side did not show any signs of osteolysis, although clinical impression was consistent with arthritic changes over the acetabular side of the left hip. It was recommended that she undergo conversion to a total hip arthroplasty. This was performed in June 2010 using a 48 mm Stryker Trident PSL acetabular component with an X3 28 mm liner and L-fit 28 mm head (Figure 4).

**Figure 5:**
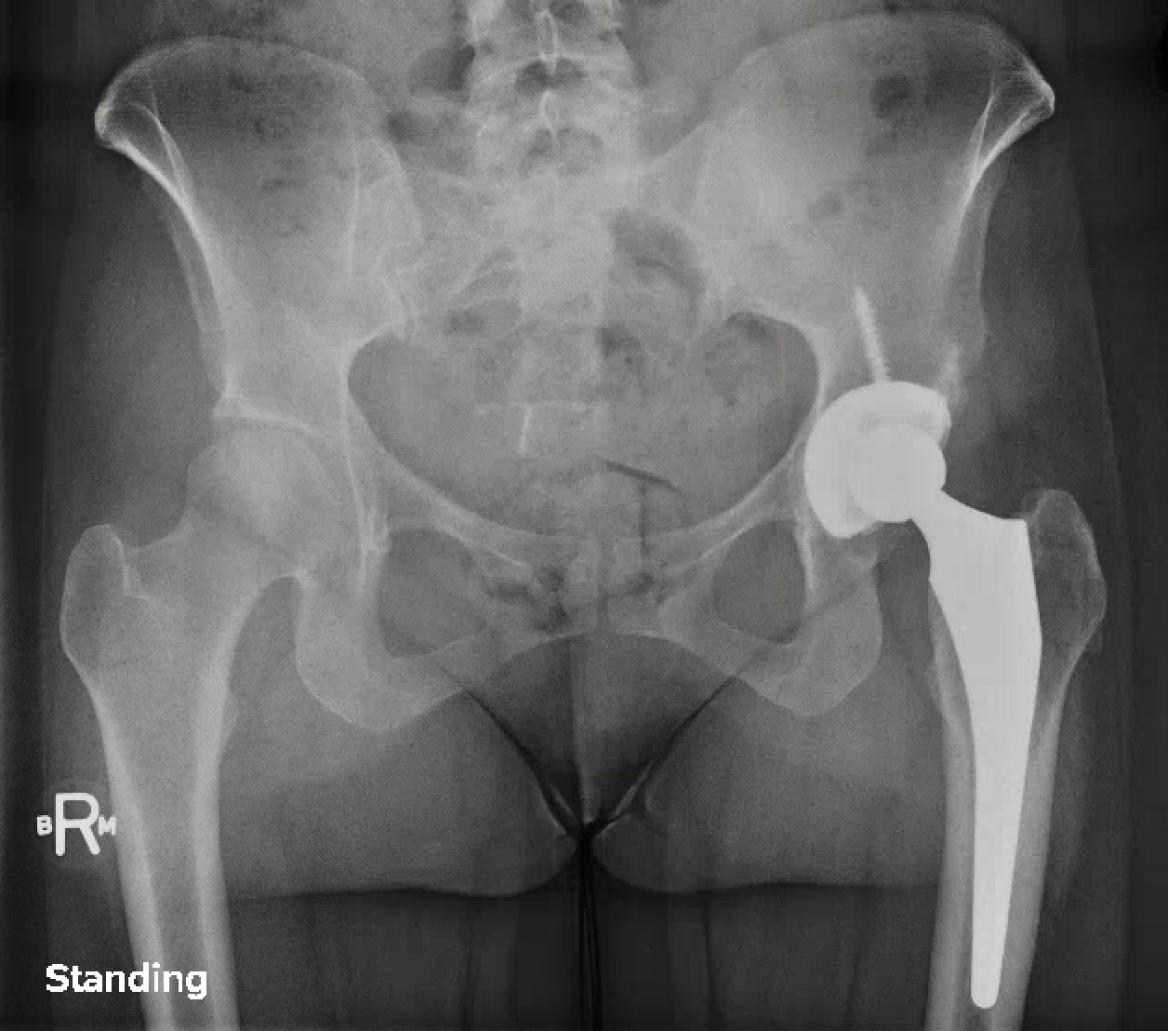
Pelvic radiograph taken at 5-years post-operatively well-aligned left total hip arthroplasty .

**Figure 6:**
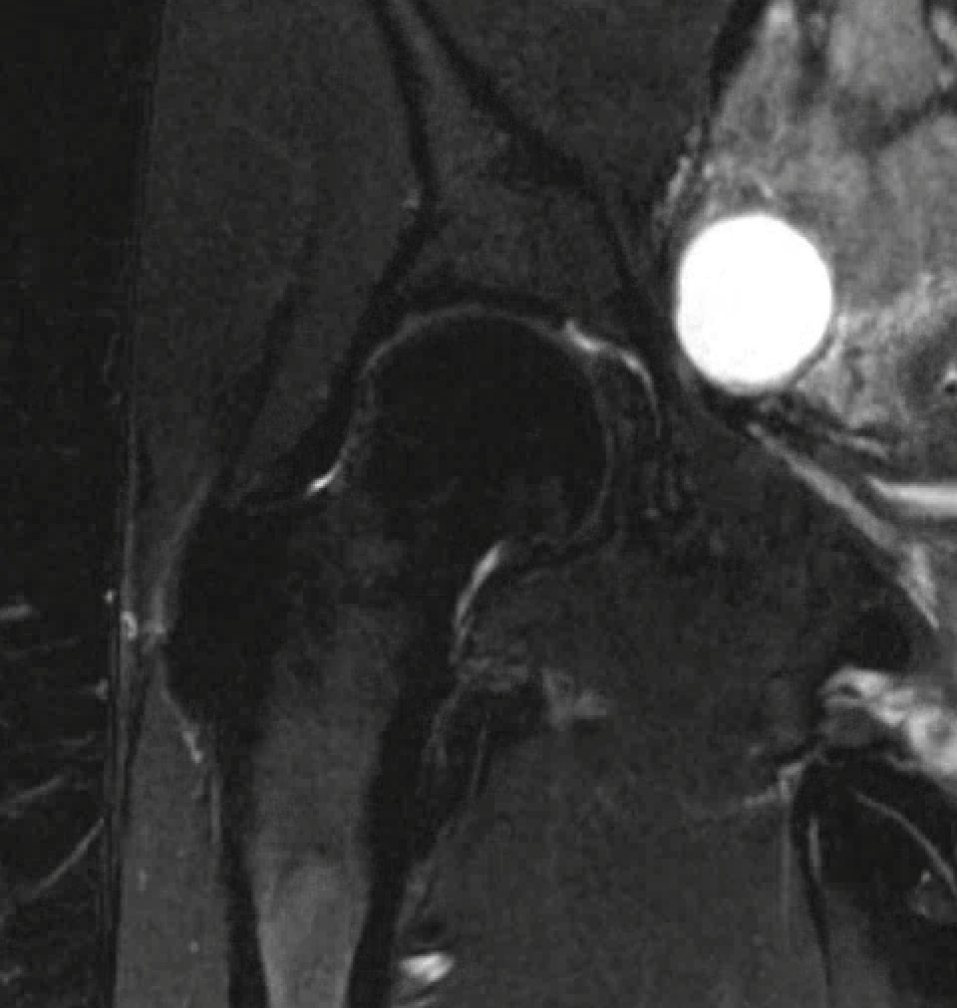
Coronal T2-weighted magnetic resonance image of right hip showing preservation of joint space and no evidence of femoral head collapse.

Since her total hip arthroplasty, she has experienced significant improvement in her hip pain bilaterally. Radiographs taken in 2015 at her 5-year follow-up demonstrated the components to be in proper position on the left side (Figure 5) and preserved femoral head shape. Minimal degenerative changes in the right hip with no advancement of collapse were seen on MRI performed at the same visit (Figure 6).

## Discussion

Hip pain during the later stages of pregnancy and during the postpartum period is a common presentation and is usually related to sciatica, pelvic structural compression, and lumbosacral strain [9]. Transient osteoporosis, which is self-limiting and typically resolves within months, and osteonecrosis, which results in femoral head collapse and degenerative changes in the joint, can also cause pain [4,9,13]. The use of MRI is an effective method for evaluating patients with osteonecrosis of the femoral head during pregnancy [9,13] since the early stages of osteonecrosis are not easily recognized clinically during antenatal and postnatal care [14].

The etiology of osteonecrosis of the femoral head during pregnancy is still largely unknown. However, theories have been proposed suggesting the pathogenesis is likely to be multifactorial including hormonal, mechanical, and coagulation factors [4,11].

 Venous congestion and hypercoagulability are common in the third trimester during pregnancy. Osteonecrosis is more common on the left side than the right side in pregnant women, which can be explained by the anatomy of venous drainage [4].  The left common iliac vein passes deep to the right common artery and may be subject to excessive compression from the weight of the developing fetus [4,11].

Other possible etiologies are ovarian hyperstimulation drugs, which have the detrimental effects of hyperviscosity and hypercoagulability [10-11], and the mechanical stress or overload by excessive labor and weight gain during the last trimester of pregnancy [9,13].

Furthermore, many endocrine modifications occur during pregnancy such as parathyroid hyperplasia and the production of estrogen and progesterone by the placenta [13]. These can destabilize endogenous plasma lipoproteins and lipid metabolism in the liver, which could promote fat embolism [4,9,11,15].  Increases in estrogen and progesterone results in increased adrenocortical activity and levels of adrenal corticosteroids to almost three times the level of a non-pregnant woman [9, 11,13,15].

The available literature is scarce with regard to the single or dual dosage of steroids that will cause osteonecrosis. Zhang et al., reviewed 43 cases of steroid-induced osteonecrosis following the SARS epidemic and suggested that a single dose of 200 mg of methylprednisone or a cumulative dose of more than 4000 mg was a significant risk factor for the development of multifocal osteonecrosis [7]. Gunal and Karatosun showed bilateral osteonecrosis of the hip after a single dose (75.5 mg) for treatment of an allergic reaction [6]. Mckee et al., reviewed 15 cases of osteonecrosis with a mean of 20.5 days of treatment and doses of up to 3300 mg of prednisone [5]. Still, there is no widely accepted critical dosage or duration of steroid treatment that is thought to cause osteonecrosis because it is difficult to conclusively establish causality. The above presented case suggests that an even lower threshold may be associated with development of the condition.

The optimal treatment of osteonecrosis in a young patient is controversial. Conservative methods are usually initiated when there is no evidence of collapse such as restricted weight bearing [4].  Furthermore, many surgical options have been proposed according to the stage of the disease.  These include core decompressions, vascularized and non-vascularized grafts, osteotomy, and as a last resort option arthroplasty [1,4,9].  Vascularized structural grafting has been shown to be helpful in the prevention of articular collapse, thus delaying the need for total hip arthroplasty [4]. However, at this time the most frequently advocated treatment for osteonecrosis of the femoral head with evidence of collapse is total hip arthroplasty [1].  The drawback of such a treatment in younger patients is the lack of options when the implants fail [1,4]. Total hip arthroplasty was performed in our patient, and she had notable pain relief and functional improvement.

## Conclusions

In summary, although a rare occurrence, development of osteonecrosis after pregnancy (with or without steroid administration) should be high on the differential for postpartum hip pain. This will help practitioners potentially identify disease at an earlier stage before femoral head collapse occurs, which may allow for joint preservation as opposed to replacement.
